# The relationship between mobile phone addiction and physical activity behavior among university students: the mediating role of bedtime procrastination

**DOI:** 10.3389/fpsyg.2025.1603046

**Published:** 2025-07-30

**Authors:** Wanbin Yu, Xielin Zhou, Bo Li

**Affiliations:** ^1^Department of Physical Education, Chengdu Sport University, Chengdu, Sichuan, China; ^2^Department of Sports Training, Chengdu Sport University, Chengdu, Sichuan, China

**Keywords:** mobile phone addiction, physical activity behavior, bedtime procrastination, university students, mediating role

## Abstract

**Purpose:**

To explore the relationship between mobile phone addiction and physical exercise behavior among college students, and to analyze the mediating effect of bedtime delay between the two, to provide a basis for the development of good behavioral habits among college students.

**Methods:**

The Mobile Phone Addiction Scale, the Physical Activity Behavior Self-Assessment Scale, and the Bedtime Procrastination Scale were administered to 356 college students (49.4% male) in Sichuan Province. Structural equation modeling was then conducted to test the mediating effects.

**Results:**

(1) There is no gender difference in mobile phone addiction, bedtime procrastination, and physical activity behavior among college students (all *p* > 0.05). (2) There was a significant negative correlation between college students‘ mobile phone addiction and physical activity behavior (*r* = −0.688, *p* < 0.01); a significant positive correlation between college students' mobile phone addiction and bedtime delay (*r* = 0.730, *p* < 0.01); and a significant negative correlation between bedtime delay and physical activity behavior (*r* = −0.658, *p* < 0.01). (3) Bedtime delay mediates the relationship between mobile phone addiction and physical activity behavior among college students (β = −0.27).

**Conclusion:**

(1) There is a close relationship between mobile phone addiction, bedtime procrastination, and physical activity behavior among college students; (2) Mobile phone addiction significantly impacts the physical activity behavior of college students. Additionally, it indirectly influences their physical activity behavior through procrastination at bedtime.

## 1 Introduction

Physical activity behavior refers to the physical activities individuals engage in to enhance their health, improve motor skills, and promote positive exercise habits (Mercier et al., 2023). These activities can reflect individuals' values toward sports, physical fitness, and mental wellbeing, making them an essential component of daily life (Lu and Baolin, 2017). Although colleges and universities have formulated a series of programs to improve college students' participation in physical exercise, according to data from an exercise survey, the proportion of college students who exercise less than three times a week is as high as 48.19, and 58.7% of college students do not exercise more than 30 min at a time (Daily, 2023). Several studies have identified academic pressure, disinterest in sports, and inadequate equipment and facilities as the primary factors contributing to the low levels of physical activity among college students in China (Yun et al., 2019). In recent years, researchers investigating the attribution of physical activity behaviors have discovered a correlation between mobile phone addiction, sleep quality, and physical activity behaviors in college students (Yang et al., 2023; Zhao and Kou, 2024), in which bedtime procrastination affects the quality of individual's sleep, which manifests itself in the fact that the worse the bedtime procrastination behavior, the worse the quality of sleep (Hill et al., 2022). Bedtime procrastination is characterized by a significant delay between the intended bedtime and the actual time an individual falls asleep, indicating a failure in self-regulation (Liu et al., 2024). Mobile phone addiction, on the other hand, refers to a pattern of behavior in which an individual uses his or her mobile phone excessively and becomes dependent on it, affecting daily life, work, study and interpersonal relationships, etc., and is a manifestation of low self-control (Chen et al., 2024; Liu et al., 2022). Both phenomena not only diminish the sleep quality of college students and weaken the body's immune system (Zhao et al., 2024), but also damage the cervical spine and affect normal life (Gao et al., 2018). Therefore, exploring the interactive effects of mobile phone addiction and bedtime procrastination on physical activity behaviors of college students is necessary to promote the physical and mental health development of college students and to cultivate good physical activity behaviors among college students.

Self-determination theory (SDT) suggests that individuals have three basic psychological needs: autonomy, competence, and relatedness. When college students are continuously frustrated or unable to satisfy these needs in real life, they may turn to their mobile phones for compensatory satisfaction, resulting in mobile phone addiction (Flanigan et al., 2023). Self-determination theory (SDT) suggests that individuals have three basic psychological needs: autonomy, competence, and relatedness. When college students are continuously frustrated or unable to satisfy these needs in real life, they may turn to their mobile phones for compensatory satisfaction, resulting in mobile phone addiction (Niu, 2023). Mobile phone addicts tend to overindulge in virtual gratification, resulting in significant crowding out of time and energy devoted to other important real-world activities, such as physical activity. It has been further demonstrated that mobile phone addiction negatively affects an individual's physical exercise activity (Kim et al., 2015). That is, the more time an individual spends using a mobile phone, the lower the physical activity commitment is (Pereira et al., 2020). Physical exercise is an important way for individuals to enhance their physical fitness, enjoy themselves physically and mentally, and promote their mental development (Zhang et al., 2022). Long-term lack of physical activity can trigger muscle atrophy and respiratory, circulatory system decline, causing low back pain, arthralgia, and other symptoms, producing obesity, cardiovascular disease, osteoporosis, and other hazards (Wojcicki et al., 2013). This shows that the importance of physical activity is unquestionable, and mobile phone addiction decreases physical activity. Therefore, this study proposes hypothesis H1: Mobile phone addiction is negatively associated with physical activity behavior.

The convenience of mobile phones makes individuals more susceptible to addictive behaviors and affects their ability to self-regulate, leading to procrastination (Shi et al., 2021). According to a survey conducted by the website Procrastination and Science, between 80 and 95% of college students worldwide agree that they procrastinate (Daily, 2019). According to Self-Determination Theory (SDT), when individuals turn to mobile phones for compensation for unmet basic needs, their ability to self-regulate their behavior (including mobile phone use itself) may be weakened, manifesting as low self-control. This fits highly with the self-regulatory failure theory of procrastination. This theory suggests that bedtime procrastination is essentially the result of a failure of self-regulation between an individual's intentions (e.g., to go to bed on time) and their actual actions, and centers on the triumph of immediate gratification preferences over long-term goal orientation, reflecting the depletion of, or lack of, self-control resources (Magalhaes et al., 2021). The Internet displacement hypothesis further elucidates that when individuals spend too much time on mobile phone use, they have less energy for other activities and sleep activities, which creates procrastination in related activities (bedtime procrastination; Kadzikowska-Wrzosek, 2020; Ma et al., 2024). Mobile phone addiction-induced pre-bedtime mobile phone use behavior gradually causes individuals to delay bedtime (Feng and Sun, 2023; Lian et al., 2021). From the above theories, an individual's mobile phone addiction may cause the individual to fail to self-regulate at bedtime, resulting in bedtime procrastination. Therefore, this study proposes hypothesis H2a: mobile phone addiction can positively associate with bedtime procrastination.

**Figure 1:**
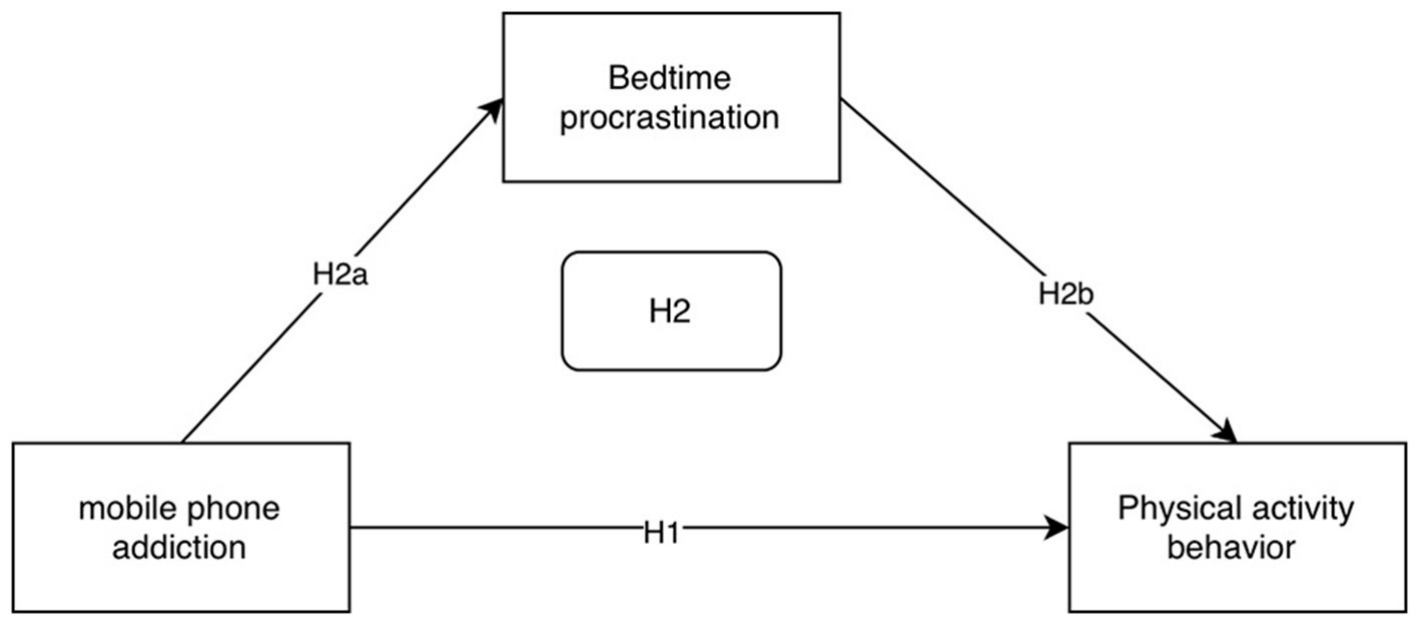
Conceptual architecture model.

Studies have found a strong link between physical activity behavior and procrastination; however, most of the studies show a unidirectional dimension in their conclusions, i.e., physical activity behavior reduces procrastination (Shi et al., 2021), but whether procrastination behaviors (e.g., bedtime procrastination) can affect physical activity behaviors needs to be further explored. In studying the relationship between physical activity behavior and sleep quality, previous researchers have mainly focused on the role of physical activity on sleep quality and found that good physical activity behavior can improve the sleep quality of individuals (Castelli et al., 2023; Hachenberger et al., 2023; Merellano-Navarro et al., 2022). So, does bedtime delay negatively affect physical activity? The Theory of Planned Behavior explains the relationship between an individual's behavior and intentions in terms of the theory, which suggests that intentions are a direct determinant of behavior, which is influenced by behavioral attitudes and subjective norms. Chinese scholars have explained college students‘ procrastination based on the Theory of Planned Behavior (TPB) and proposed the idea that there is an association between TPB and procrastination (Lin, 2014). At the same time, there is an association between physical activity behavior and the theory of planned behavior, i.e., physical activity behavior is also influenced by the theory of planned behavior (Zhang et al., 2021a), Then bedtime procrastination and physical activity behavior do not affect individuals individually, and there may be an association between the two. It has been further suggested that sleep quality problems caused by sleep delays can lead to sleep disorders that interfere with college students' daily behaviors (e.g., physical activity behaviors; Glavin et al., 2021). For example, bedtime procrastination causes individuals to spend more time on electronic devices such as mobile phones during the night, which in turn compresses sleep time. Sleep deprivation triggers an increase in fatigue, and the rise in fatigue in turn diminishes the individual's motivation to engage in physical activity. This chain reaction from sleep deprivation to increased fatigue to decreased motivation ultimately leads to a decrease in an individual's physical activity. Based on this, this study proposed hypothesis H2b: bedtime procrastination may be negatively associated with physical activity behavior; and hypothesis H2: the mediating role of bedtime procrastination between mobile phone addiction and physical activity behavior (Figure 1).

## 2 Materials and methods

### 2.1 Participants

This study selects students from three universities in Sichuan Province as the survey object through convenience sampling, and the survey time is from 29 June 2024 to 2 July 2024 (from 29/6/2024 to 2/7/2024), in order to ensure the subjects' right to know and follow the principle of voluntariness, the confidentiality programme of the content filled in by the subjects was informed before the administration of the test, and 433 questionnaires were distributed through the questionnaire star platform at the time of the survey after the consent was given, and 356 valid questionnaires were recovered with an effective recovery rate of 82.2%. Among them, the number of male students was 176, accounting for 49.4 %, and the number of female students was 180, accounting for 50.6%.

### 2.2 Methods

#### 2.2.1 Mobile phone addiction scale for college students

The Smartphone Addiction Scale for College Students compiled by Su et al. (2014), which adopts a 5-point Likert scale, with 5 levels for each entry (1 = very non-compliant; 2 = non-compliant; 3 = unsure; 4 = compliant; 5 = very compliant), and has a total of 22 entries containing 6 factors, including withdrawal, salience, social interaction, pacification, negative influence, app use, app update, and the higher score represents the more serious mobile phone addiction. Pacification, Negative influence, App use, App update 6 factors, the higher the score represents the more serious mobile phone addiction, validation factor analysis obtained on the 6-factor conceptual structure of the various good fit indicators proves that the scale has good structural validity. The Cronbach α coefficient in this study was 0.936.

#### 2.2.2 Bedtime procrastination scale

The scale was adopted for the Chinese version of the Bedtime Procrastination Scale compiled by Kroese and revised by Zhang et al. (2021b), which was mainly for college students, with 9 entries, using a 5-point Likert scale (1 = never; 2 = occasionally; 3 = sometimes; 4 = frequently; and 5 = always), with 4 of the entries being reverse-scored, with a higher score on the scale indicating a greater tendency to procrastinate bedtime, and with a high Cronbach α coefficient of 0.835 on its re-test reliability. The Cronbach α coefficient in their study was 0.835 and the retest reliability was 0.72, possessing a high validity scale validity. The Cronbach α coefficient of this study was 0.872.

#### 2.2.3 Physical activity behavior scale

The scale of college students' physical activity behavior was based on the physical activity behavior subscale of the Physical Activity Rating Scale compiled by Liang Deqing et al. (Qing, 1994), with a total of 8 questions and a 5-point Likert scale, with 5 ratings for each entry (1 = Very Non-compliant; 2 = Noncompliant; 3 = Uncertain; 4 = Compliant; 5 = Very Compliant), and the higher the score represented the higher the level of physical activity, the subscale questionnaire Reliability was good and the Cronbach alpha coefficient in this study was 0.949.

#### 2.2.4 Data analysis

In this study, the collected data were processed and analyzed using SPSS 27.0 and Process v3.5 by Hayes, common method bias test, descriptive statistics, correlation analysis, independent samples t-test, and ANOVA by SPSS; mediation effect analysis was conducted by using Process v3.5 by Hayes, and the model chosen was Model 4. Where M = bedtime delay, X = mobile phone addiction, Y = physical activity behavior, Bootstrap Samples = 2000.

## 3 Results

### 3.1 Common methodology bias test

The Harman one-factor test was used in this study to test for common method bias (Zhou Hao, 2004). As a result, a total of four factors with an eigenroot greater than 1 were extracted, of which the first factor cumulatively explained 24.8% of the total variance, which is less than the 40% criterion, indicating that there is no serious common method bias in this study (Podsakoff et al., 2003).

### 3.2 Descriptive statistics and correlation analysis

The current study focused on examining the overall scores of the variables in the model, no longer examining the subdimensions of the variables, and using the mean scores of the variables for Pearson correlation analysis. The results of descriptive statistics showed (as shown in Table 1) that mobile phone addiction was negatively correlated with physical activity behavior, bedtime procrastination was negatively correlated with physical activity behavior, and mobile phone addiction was positively correlated with bedtime procrastination. In this study, independent samples *t*-tests were conducted on subjects' gender and academic level, and there were no significant differences (*P* > 0.05) in gender and academic level for all three variables.

**Table 1:** Mean, standard deviation and correlation coefficient between variables.

	**M ±SD**	**1**	**2**	**3**
1.Mobile phone addiction	3.34 ± 0.75	1		
2.Bedtime procrastination	3.29 ± 0.87	0.730^**^	1	
3. physical activity behavior	2.66 ± 0.84	−0.688^**^	−0.658^**^	1

^*^p < 0.05.

^**^p < 0.01.

### 3.3 Independent samples *t*-test and ANOVA

In this study, an independent samples t-test was conducted on the gender of the subjects (as shown in Table 2), and Levene's variance equivalence test for gender showed that there was no significant difference (*P* > 0.05) between mobile phone addiction, bedtime procrastination, and physical activity behavior, and therefore, variance chi-squared data were used. In the mean equivalence t-test, there was no significant difference (*P* > 0.05) in the three variables, indicating that there is no gender difference in mobile phone addiction, bedtime procrastination and physical activity behavior among university students.

**Table 2:** Independent samples *t*-test for gender.

			**Levine's test for equality of variances**		**Mean equivalence** ***t*****-test**	
**Grouping variable**	**Implicit variable**	**HV-test**	***F***	***P***	***t***	***P***
Gender	Mobile phone addiction	Variance chi-square	0.917	0.339	−0.433	0.665
	Bedtime procrastination	Variance chi-square	0.562	0.454	−0.199	0.842
	Physical activity behavior	Variance chi-square	0.515	0.473	−0.189	0.851

ANOVA was also performed on grade and academic section (as shown in Table 3), and there was no significant difference (*P* > 0.05) in mobile phone addiction, bedtime procrastination and physical activity behavior by grade and age, indicating that there is no difference in mobile phone addiction, bedtime procrastination and physical activity behavior among university students by age and academic section.

**Table 3:** ANOVA for grade and age.

**Grouping variable**	**Implicit variable**	**Mean square**	***F***	***P***
Age	Mobile phone addiction	0.791	1.425	0.233
	Bedtime procrastination	0.788	1.035	0.31
	Physical activity behavior	1.611	2.314	0.129
Grade	Mobile phone addiction	0.186	0.333	0.717
	Bedtime procrastination	0.256	0.334	0.716
	Physical activity behavior	0.109	0.156	0.856

### 3.4 The mediating effect of bedtime procrastination on the relationship between mobile phone addiction and physical activity behavior among university students

**Figure 2:**
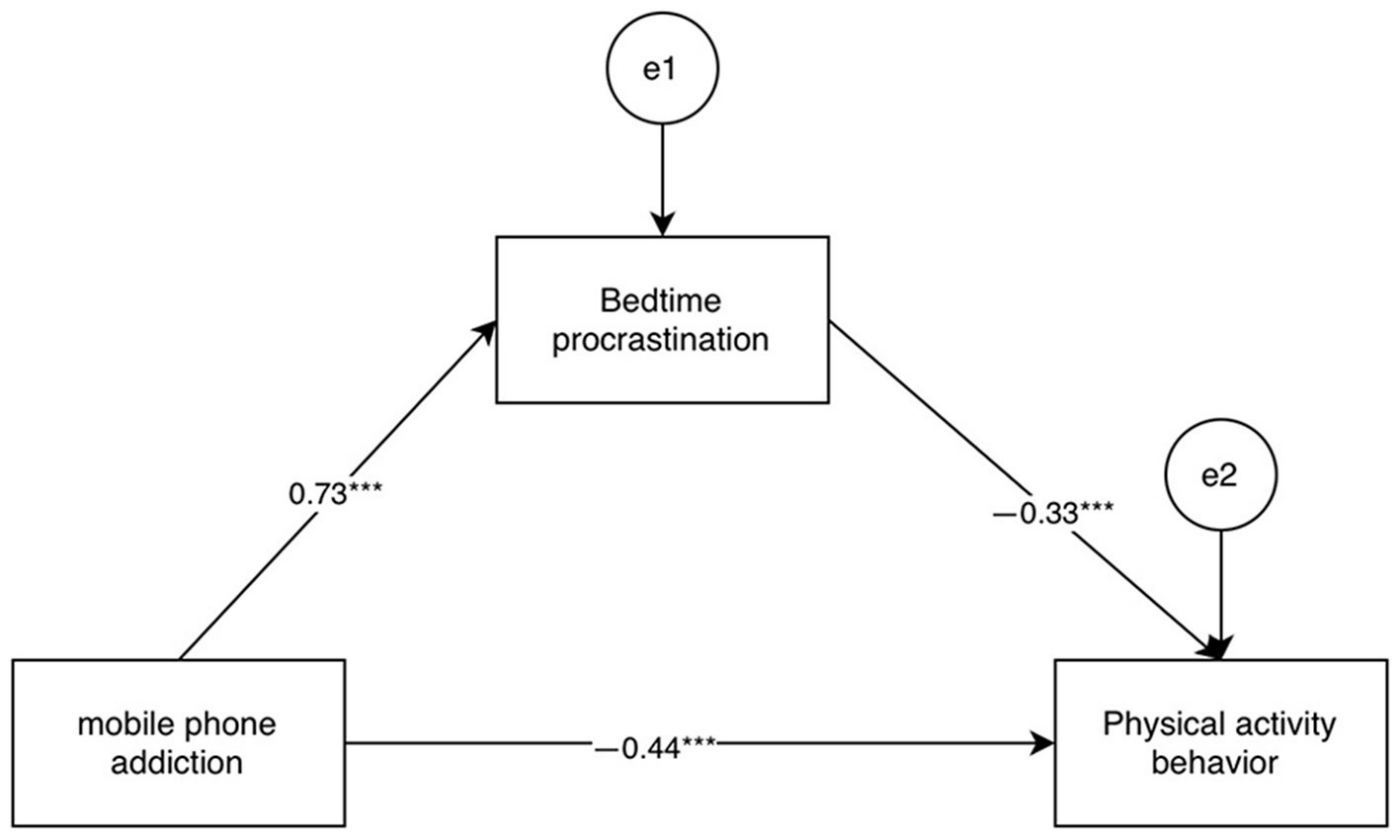
Pathways of mobile phone addiction on physical activity behavior.

College students‘ mobile phone addiction, bedtime procrastination and physical activity behavior are significantly correlated between the two, which meets the statistical requirements of the direct and indirect effect analysis of mobile phone addiction and physical activity behavior, using to carry out mediation effect analysis, select Model4, using bootstrap method to test and analyze the mediation role of bedtime procrastination in the relationship between college students' mobile phone addiction and physical activity behavior, the variable The path values between are shown in Figure 2.

The mediating effect of bedtime delay in college students' mobile phone addiction and physical activity behavior was analyzed using the bootstrap method (Table 4), with a sample size of 2,000 selected and 95% confidence intervals used, and the results showed that none of the three paths had effect values with 95% CIs that passed through 0, indicating the existence of statistical significance, with an indirect effect value of −0.27, and a mediating effect with a bootstrap 95% confidence interval of [−0.37, −0.18], accounting for 35.1% of the total effect (−0.77) of college students' mobile phone addiction on physical activity behavior (the mediation ratio is calculated as: indirect effect divided by the total effect times 100%). This result suggests that bedtime procrastination is one of the important mechanisms by which mobile phone addiction affects physical activity behavior, playing a partially mediating role, and implies that mobile phone addiction both indirectly impairs exercise behavior by disrupting sleep rhythms (bedtime procrastination). This finding deepens the understanding of the mechanisms by which mobile phone addiction affects health behaviors, empirically confirms the important role of sleep rhythm disruption (bedtime procrastination) and complements the existing multi-path theoretical framework on the negative health consequences of mobile phone addiction.

**Table 4:** Explanatory table for total, direct and indirect effects.

	**Effect value**	**SE**	**LLCI**	**ULCI**	**Effective quantity**
Total effect	−0.77	0.04	−0.86	−0.69	
Direct effect	−0.50	0.06	−0.62	−0.38	64.9%
Indirect effect	−0.27	0.05	−0.37	−0.18	35.1%

## 4 Discussion

This study reveals the direct and indirect paths of mobile phone addiction in the process of influencing college students' physical activity behavior, which is a positive exploration of preventing college students' excessive use of mobile phones, improving their participation in physical activity, and promoting their physical and mental health. At the theoretical level, this study enriches the research on the factors and mechanisms influencing college students' physical exercise behaviors and mental health, and deepens the research results of physical education; at the practical level, it suggests that mobile phone addiction and bedtime procrastination both belong to the bad life habits, as well as their influences on the physical exercise behaviors, and it provides a new way of thinking for the promotion of the development of good habits among college students.

### 4.1 Direct effects of mobile phone addiction on physical activity behavior among university students

The results of the current study showed that mobile phone addiction among college students was negatively associated with physical activity behavior, and hypothesis H1 was confirmed. As Internet technology continues to advance, smartphones are playing an increasingly crucial role in individuals' lives. For college students, smartphones are indispensable for social interaction, entertainment, shopping, and payment (Lee et al., 2017). However, the growing usage of mobile phones among college students has led to an escalation in dependency, making it challenging for them to exercise self-control as they engage in excessive Internet browsing and watching short videos. This behavior has significant repercussions on both their physical and mental wellbeing (Stuckey et al., 2017). According to the theory of rational behavior, an individual's behavioral intention directly influences their actions, while behavioral attitudes and subjective norms impact these intentions (Bleakley and Hennessy, 2012). Consequently, when college students grapple with mobile phone addiction, their intentions tend to lean toward increased mobile phone usage. This is evident in their heightened focus on mobile phone usage in daily life, ultimately resulting in a substantial impact on their leisure time. Use, which leads to the spare time being occupied by head-down behavior and screen-front behavior, and physical exercise is naturally difficult to guarantee (Salehan and Negahban, 2013), which not only affects the physical health of college students but may also harm their study and life. It is also mentioned in the internet displacement hypothesis that when individuals spend too much time on mobile phone use, the less energy they put into other activities (Ma et al., 2024). This study demonstrates the detrimental impact of mobile phone addiction on the physical activity behavior of college students. The findings indicate that as the level of addiction increases, the attitude toward exercise becomes more negative. Additionally, it has been established that mobile phone addiction can result in the development of negative emotions, ultimately leading to a state of physical inactivity in individuals (Gong et al., 2023). This can explain the relationship between mobile phone addiction and physical activity behavior, and the results of this study further confirm that mobile phone addiction reduces individuals' physical activity behavior, which is consistent with previous studies (Zagalaz-Sánchez et al., 2019), and enriches the existing studies while providing theoretical and practical insights into intervening in college students' physical activity behavior: i.e., it can be achieved by improving the individual's self-control ability, improving mobile phone addictive habits, restraining the frequent head-down and screen-front behaviors, thus promoting the physical and mental health development of college students.

### 4.2 The mediating role of bedtime procrastination

The results of the mediation effect show that, on the one hand, college students' mobile phone addiction is negatively associated with physical activity behavior, which indicates that to improve the physical and mental health of college students, it is necessary to reduce the time of college students' mobile phone use, and then improve the level of physical activity. On the other hand, college students' mobile phone addiction can also be indirectly associated with physical exercise behavior through bedtime procrastination, and bedtime procrastination plays a mediating role between college students' mobile phone addiction and physical exercise behavior, which is manifested in the following way: college students' mobile phone addiction is positively associated with bedtime procrastination (H2a), and bedtime procrastination is negatively associated with physical exercise behavior (H2b), which can be seen in this way, hypothesis H2 is valid. Mobile phone addiction, identified as a novel behavioral addiction phenomenon, has the potential to negatively impact both the physical activity and mental wellbeing of individuals (Xiang et al., 2023). Research has shown that mobile phone addiction can lead to procrastination tendencies (Márquez-Hernández et al., 2020). This addiction is characterized by the excessive and uncontrolled use of mobile phones (Tong and Meng, 2023), which can diminish an individual's self-regulation toward mobile phone usage, leading to feelings of anxiety, depression, and subsequent procrastination behaviors (Yang Xiaofan and Hu, 2020). This study contributes to the existing literature by establishing a positive correlation between mobile phone addiction and bedtime procrastination. Furthermore, it suggests that the severity of bedtime procrastination is directly proportional to the level of mobile phone addiction. Moreover, procrastination has been found to influence individuals' motivation to engage in physical activities (Tao et al., 2024), according to the temporal motivation theory, procrastination arises from a lack of self-regulation, which in turn is linked to individuals' inability to control their behavior (You et al., 2020). Individuals with low self-control are less likely to adhere to physical activity routines (Hagger et al., 2019), indicating that procrastination hinders their participation in physical exercise. Some scholars have posited that individuals who engage in moderate-intensity physical activity regularly must possess a high level of time management skills. On the other hand, those who procrastinate going to bed demonstrate a lack of time management ability, as they struggle to regulate their bedtimes effectively. This lack of time management is linked to a significant and negative impact on physical activity behavior (Schöndube et al., 2017). Moreover, the study suggests that bedtime procrastination acts as a mediator between mobile phone addiction and physical activity behaviors among university students, as indicated by the positive association between mobile phone addiction and bedtime procrastination, and the negative association between bedtime procrastination and physical activity behaviors, in line with previous research findings.

### 4.3 Limitations of the study

This study explored the effects of mobile phone addiction on physical activity behavior among college students and revealed the mediating role of bedtime delay in mobile phone addiction and physical activity behavior, but there are still some shortcomings, and future research can be improved in the following aspects:

(1) This study used a cross-sectional design where data were collected at the same point in time. Although the mediating effect was tested through the Bootstrap method and the directionality between the variables was assumed based on theory, this design could not rigorously establish the causal relationship between the variables. Therefore, future research could use a longitudinal tracer study or experimental intervention design to verify the causal time series between these variables.
(2) The sample for this study originated from only one province, and although the sample size fulfills the basic conditions of statistics, there are limitations in the sample size in terms of geographical distribution, types of schools, and composition of majors. The results of the study may not apply to regions outside of Sichuan Province. Future studies may consider the possible systematic differences in mobile phone usage habits, academic pressure, lifestyles, and attitudes toward physical activity among college students from different geographic regions, different types of colleges, and different professional backgrounds.
(3) The demographic variables in this study included gender, age, and grade level, but some potential factors (e.g., social activity patterns, campus athletic facilities, and personal interest preferences) still existed that could have an impact on mobile phone addiction, bedtime procrastination, and exercise behavior. Future research could more systematically identify and incorporate these potentially important covariates for analysis.

## 5 Conclusion

This study proves the close relationship between college students' mobile phone addiction, bedtime procrastination and physical activity behavior, which is manifested in the fact that college students' mobile phone addiction can affect physical activity behavior, and at the same time, it can indirectly affect college students' physical activity behavior through the path of bedtime procrastination. Among them, sleep delay aggravates the harm of mobile phone addiction on physical exercise behavior, which is not conducive to the physical and mental health development of college students.

Therefore, based on the findings of the study, colleges and universities should adopt diversified and specific intervention strategies to put attention to college students' life patterns, mobile phone use habits, and physical exercise into practice. Research Recommendation:

(1) Colleges and universities have launched a series of health education and skills training programmes, incorporating healthy work and rest, time management, scientific mobile phone use skills and the benefits of exercise into the education system for new students, the general education curriculum, or the mental health education curriculum. At the same time, mobile phone screen time management APPs and exercise punch cards APPs are promoted on campus to help students record and plan exercise.
(2) Colleges and universities optimize the physical education curriculum, adjust the content of physical education courses, and increase the number of interesting and diversified sports, such as yoga, street dance, rock climbing, and outdoor development, to meet the interests of different students. At the same time, provide personalized physical education course choices, allowing students to choose suitable courses according to their own physical condition and interests.
(3) Colleges and universities build a multi-departmental collaborative mechanism consisting of the Office of Student Affairs, the Academic Affairs Office, the Physical Education Department, the Mental Health Centre, and other departments to jointly develop and implement intervention plans. Each department has a clear division of labor and collaborates to promote the management of mobile phone use and the promotion of physical activity.

## Data Availability

The original contributions presented in the study are included in the article/supplementary material, further inquiries can be directed to the corresponding author.
